# Non-invasive brain stimulation in early rehabilitation after stroke

**Published:** 2015

**Authors:** AV Blesneag, L Popa, AD Stan

**Affiliations:** * Department of Clinical Neurosciences, ‘‘Iuliu Hațieganu’’ University of Medicine and Pharmacy, Cluj-Napoca, Romania; ‘‘RoNeuro’’ Institute for Neurological Research and Diagnostic, Cluj-Napoca, Romania

**Keywords:** stroke, neurorehabilitation, transcranial direct current stimulation, repetitive transcranial magnetic stimulation

## Abstract

The new tendency in rehabilitation involves non-invasive tools that, if applied early after stroke, promote neurorecovery. Repetitive transcranial magnetic stimulation and transcranial direct current stimulation may correct the disruption of cortical excitability and effectively contribute to the restoration of movement and speech. The present paper analyses the results of non-invasive brain stimulation (NIBS) trials, highlighting different aspects related to the repetitive transcranial magnetic stimulation frequency, transcranial direct current stimulation polarity, the period and stimulation places in acute and subacute ischemic strokes. The risk of adverse events, the association with motor or language recovery specific training, and the cumulative positive effect evaluation are also discussed.

**Abbreviations:** AAT = Aachen Aphasia Test, BDNF = brain-derived neurotrophic factor, IFG = inferior frontal gyrus, M1 = primary motor cortex, MRI = magnetic resonance imaging, NIBS = non-invasive brain stimulation, PET = positron emission tomography, rTMS = repetitive transcranial magnetic stimulation, SLT = speech and language therapy, STG = superior temporal gyrus, tDCS = transcranial direct current stimulation, NIHSS = National Institutes of Health Stroke Scale

## Cortical reorganization after stroke

Due to its intrinsic plasticity, the brain has the capacity to restructure its neural networks and to reconfigure its activity after an ischemic or haemorrhagic stroke. The metabolic and functional cerebral alteration can be observed by functional imaging studies (positron emission tomography (PET) scan and functional magnetic resonance imaging (fMRI), and also by transcranial magnetic stimulation mapping of the motor area [**1**,**2**]. After an injury, there are changes in motor and sensitive cortical somatotopies, both in the damaged region and the remote areas from ipsi- and contralateral hemisphere. An increased activation in contralesional primary motor cortex (M1), bilateral ventral premotor cortex and supplementary motor area are commonly found in stroke patients [**3**]. While the healthy subjects show greater activity in the contralateral hemisphere to the moving hand, stroke patients assume a bilateral pattern in general [**4**]. 

In the recovery of aphasia, three models of neuroplasticity were framed by prior research: (1) activation of perilesional language areas in the dominant hemisphere, (2) recruitment of homologue area in the non-dominant hemisphere, and (3) improper recruitment of different regions in the non-dominant hemisphere (maladaptive plasticity) [**5**]. These mechanisms are activated differently depending on stroke size and location. Small ischemic or haemorrhagic lesions may be compensated by the recruitment of residual language areas and the increase in perilesional activity. By contrast, the larger lesions in the language regions are followed by the recruitment of homotopic non-dominant areas [**6**]. 

However, the role of right language homologues in the early recovery after stroke seems to be transient, to an extent perilesional neuronal recruitment occurs [**5**]. Secondary to the decrease of transcallosal inhibition, some right hemispheric areas may deploy an inhibitory effect over the residual perilesional areas in the left hemisphere, interfering with the language recovery. Based on these data, a new model for the recovery of aphasia was proposed based on the suppression of non-dominant hemisphere activity and the stimulation of the perilesional areas in dominant hemisphere [**5**]. 

## Novel techniques of neuromodulation and neurorehabilitation

Transcranial direct current stimulation (tDCS) of the brain is an electrophysiological method which may be used to modulate the neocortical excitability. A direct current generator is connected to two patch electrodes (an active and a reference one) positioned over the scalp, to stimulate the subjacent tissue with low amplitudes currents (0.5 - 2.0 mA) and to modify the threshold for discharge of cortical neurons. The modulation of cortical excitability is polarity-dependent: “anodal stimulation” (increased in network excitability) – when the active electrode generates a positive potential compared to the reference electrode; “cathodal stimulation” (decreased in network excitability) – when the active electrode generates a negative potential [**4**]. 

Repetitive transcranial magnetic stimulation (rTMS) is another therapeutic tool that can contribute to the modulation and reconfiguration of different cortical areas. In contrast to tDCS, rTMS results in the induction of action potentials and depolarization of the neuronal membrane. The principle of transcranial magnetic stimulation is to produce a perpendicular magnetic field, by a stimulating coil, which induces an electric field, which, in contact with the cerebral tissue, is transmitted as an electric current, parallel to the generating coil. The depth of the stimulation depends on the type of coil and the intensity of the stimulation. The excitatory or inhibitory effect is correlated with the frequency of rTMS: high frequencies (≥ 5 Hz) are excitatory, while low frequencies (≤ 1 Hz) are inhibitory [**4**,**7**]. 

By modulating cortical excitability with respect to neural plasticity, these two non-invasive brain stimulation (NIBS) techniques represent potential therapeutic tools in recovery after stroke [**8**,**9**]. 

There are two main directions of NIBS treatment in neurorehabilitation: the motor deficit and the speech disorders. The improvement of motor and language performances is based on interhemispheric competition theory [**10**-**14**]. This model suggests that the balance between the left and the right hemisphere of stroke patients is altered by an increased interhemispheric inhibition from the unaffected hemisphere to the affected hemisphere. Accordingly, a therapeutic effect may be obtained either by increasing the excitability of the affected hemisphere, or by decreasing the excitability of the unaffected hemisphere [**10**]. The mechanism of excitability enhancement in the motor cortex underlies the motor learning and the use-dependent plasticity, which are impaired in the affected hemisphere. Other positive effects of the NIBS are the enhancement of neural coupling between the primary and secondary motor areas in the affected hemisphere, the reduction of the hyperactivity in the primary and secondary motor areas in the unaffected hemisphere, and the excitability modulation in both hemispheres [**10**].

The advantages of inhibitory NIBS over the intact hemisphere are the uniform response and the lower risk of potential epileptic seizures, which are more probably to appear after rTMS in the infarcted area [**15**]. The major disadvantage is the deterioration of bimanual movement due to the reduction of transcallosal inhibition that controls that kind of movement. This possible consequence may be prevented by applying a low frequency rTMS concomitant with anodal tDCS over the affected hemisphere [**16**]. The neural modulation induced by NIBS and the improvement of motor and speech deficits can be sustained by subsequent motor training and speech therapies [**10**,**13**].

Although it was demonstrated that the early rehabilitation facilitates motor recovery after stroke, the application of NIBS in acute stages is controversial [**17**,**18**]. The genetic polymorphism can be involved and may explain the different response of stroke patients to this therapy. BDNF is one of the factors strongly involved in synaptic plasticity of the human motor cortex [**9**]. The consequence of BDNF val66met, a common single nucleotide polymorphism, is the reduced secretion of BDNF [**19**]. This is clinically expressed by a poorer retention in short-term motor learning and slower cognitive and motor recovery [**9**,**19**]. Therewith, the individual variation in response to rTMS may be explained by the difference in BDNF concentration [**20**]. 

The majority of the clinical studies evaluating the role of rTMS in rehabilitation were performed in subacute and chronic stroke patients, few data being available for acute stroke [**21**]. In this respect, a recent Cochrane meta-analysis evaluated the efficacy and safety of rTMS in recovery after stroke regardless of the duration of illness [**22**]. Nineteen randomized controlled trials (RCTs) with a total of 588 patients were included. Even if the rTMS treatment was not associated with a significant improvement in activities of daily living, or a significant improvement of motor function, a positive trend was noted, larger and homogeneous RCTs being needed in order to confirm the results [**22**]. 

The most encountered adverse events reported in clinical trials evaluating rTMS are transient or mild headache (2.4%), anxiety (0.3%), neurocardiogenic syncope after initial exposure to rTMS (0.6%), exacerbation of pre-existing insomnia (0.3%) and local discomfort at the site of the stimulation [**22**]. Also, epileptiform discharge on EEG and seizures, can be noted especially after high-frequency rTMS (10 Hz) [**11**].

## Motor and speech recovery – rTMS studies in acute neurorehabilitation after stroke

In 2005, Khedr and colab. applied 3 Hz, in 10 daily stimulation sessions on the affected side in 26 patients with acute cortical stroke and in 26 patients with acute subcortical stroke (recruited between the fifth and tenth day post-stroke). The evaluation of motor recovery showed an improved function in the paretic hand in both groups [**23**]. 

Liepert and colab. showed that one session of 1 Hz rTMS therapy (20 minutes, 1200 pulses) on the contralesional hemisphere in 12 patients with acute subcortical stroke (mean 7 days), disclosed an enhancement in the hand functioning at Nine Hole Peg Test (a transient improved dexterity of the affected hand), but no change in the grip strength [**24**]. 

In 2010 Khedr and colab. published another study on 48 acute stroke patients (subcortical and cortical) which supported 3 Hz, 130% resting motor threshold intensity, or 10 Hz, 100% resting motor threshold intensity, in 5 daily stimulation sessions on the ipsilateral hemisphere. The improved hand function persisted for 12 months after rTMS treatment [**25**]. 

Weiduschat studied the effect of 1 Hz rTMS stimulation together with subsequent speech and language therapy (SLT) in 10 right-handed patients at 16 weeks after stroke [**26**]. Participants (5 with Wernicke’s aphasia, 2 with Broca’s, 2 with global and 1 with amnestic aphasia) were divided in two groups – the study group (5 patients) – stimulated in the right pars triangularis, and the control group (5 patients) – stimulated in vertex (sham stimulation). The results pointed out an improvement of Aachen Aphasia Test (AAT) scores in patients from the study group, but not in those with sham stimulation. Compared with baseline, a larger activation index was observed on PET scan in the right hemisphere in the control group compared with the study group, 2 weeks after rTMS, suggesting that inhibitory magnetic stimulation of the right-hemispheric Broca homologue followed by SLT might be a feasible and effective complementary therapy for post stroke aphasia [**26**]. 

Waldowski and collab. studied the effects of 1 Hz rTMS (15 days, 30 min/ day) over pars triangularis and pars opercularis from the right inferior frontal gyrus (IFG), followed by 45 min/ day SLT, in 13 right-handed patients with aphasia [**27**]. The patients were included between 2-12 weeks after the onset. This group was compared with a sham stimulation group (13 right-handed, age, sex and education-matched patients, with a first-ever left-sided middle cerebral artery subacute stroke and severe to moderate language deficits). The assessment included Computerized Picture Naming Test, Boston Diagnostic Aphasia Examination and Aphasia Severity Rating Scale. After a follow-up period of 15 weeks, no differences in naming accuracy between the two groups were noted, but reaction time was slightly faster in real rTMS group [**27**].

In a randomized, sham-controlled, double-blind study, Seniów and colab. evaluated the association of low-frequency rTMS of the contralesional M1 area with physiotherapy [**18**]. Forty patients received 3 weeks of motor training on the paretic upper limb (45 minutes daily) preceded by 30 minutes of 1 Hz rTMS (stimulated group) or by 30 minutes of sham rTMS over the contralesional M1 (control group). The functional assessment of the paretic hand (by Wolf Motor Function Test and NIHSS) performed before, immediately after and at the 3 months follow-up revealed no statistically significant differences between the two groups [**18**]. 

In 2013, Heiss et al. analyzed for the first time the consequences of the inhibitory NIBS over the contralesional IFG in left-handed aphasic patients. They reported the results of a randomized controlled trial comparing the effect of combined rTMS/ SLT in 29 right-handed patients with left hemisphere infarcts and 2 left-handed patients with right hemisphere infarcts. The start of treatment was between 25 and 93 days after stroke onset. Fifteen right-handed patients (2 with Broca’s aphasia, 8 with Wernicke’s, 3 with amnestic aphasia and 2 with global aphasia) received 10 sessions of inhibitory 1 Hz rTMS over the triangular part of the right IFG (20 min each) followed by 10 sessions of SLT, while 14 right-handers with heterogeneous aphasia forms (4 with Broca’s, 4 with Wernicke’s, 4 with amnestic and 2 with global aphasia) received 10 sessions of 20 min rTMS over the midline at the vertex (sham stimulation). Two left-handers (one with amnestic aphasia, other with Broca’s aphasia) were treated according to the same protocol with real rTMS over the left hemisphere. The speech performance was evaluated by AAT, and the language activation patterns were assessed with PET scan prior to and after the treatment. The results showed better recovery of language function in right-handed patients after real rTMS than in sham-treated right-handers. Furthermore, a shift of network activity towards the left, ipsilesional hemisphere was observed in treated right-handers, while the sham group maintained the activation in the contralesional hemisphere. Both left-handed volunteers also improved, the PET scan showing a very small interhemispheric shift (less than in rTMS-treated right-handers) and a consolidation of active networks in both hemispheres [**13**]. 

Another RCT compared the effect of 1 Hz real rTMS versus sham stimulation (10 days, 20 min/ day) over the right pars triangularis, in 24 patients suffering of subacute stroke [**12**]. In both groups, the inhibitory NIBS was followed by SLT 45 min/ day. The global AAT and naming subtests disclosed better scores, which correlated with a PET activation in the left hemisphere in rTMS group post-treatment compared to the sham group [**12**]. 

## Motor and speech recovery – tDCS studies in early rehabilitation after stroke

Hesse and colab. evaluated the role of anodal tDCS on the ipsilesional side in the recovery after stroke in 10 patients [**28**]. The tDCS was initiated 4 to 8 weeks after stroke onset. Each of the 6 daily stimulation sessions was followed by a robot-assisted arm training. Motor arm function was assessed with the Fugl-Meyer motor score and language impairment with the AAT. The motor function improved in all patients (in a significant manner in 3 cases), and aphasia improved in 4 patients [**28**]. 

In 2011, You and collab. evaluated the role of tDCS in 21 aphasic patients with subacute stroke divided in 3 equal groups: the patients in the first group received anodal tDCS on the left Wernicke’s area (superior temporal gyrus/ STG), the subjects from the second group received cathodal tDCS on the right Wernicke’s area and the other 7 participants were sham stimulated [**29**]. In all patients, the reference electrode was placed on the contralateral supraorbital region, the sessions of stimulation lasted 30 min/ day for 10 days and were completed by online SLT. The application of Korean-Western Aphasia Battery evidenced a significant improvement in auditory verbal comprehension after right cathodal tDCS compared to sham and left anodal tDCS, with an overall improvement in Aphasia Quontient and spontaneous speech across groups [**29**]. 

## Conclusion

NIBS could be a useful therapeutic tool in the early rehabilitation after stroke. Both methods may enhance the neuroplasticity in the injured area and reestablish the balance between different regions of the brain. The association with specific motor tasks and SLT reinforces the effect of NIBS and partially restores the damaged neural networks.

Larger RCTs are needed in order to confirm the effectiveness of NIBS, but the small clinical studies published so far showed encouraging results in motor and speech recovery.
